# Effect of Water Activity, pH, and Lactic Acid Bacteria to Inhibit *Escherichia coli* during Chihuahua Cheese Manufacture

**DOI:** 10.3390/foods12203751

**Published:** 2023-10-12

**Authors:** Nidia Aracely Chacón-Flores, Guadalupe Isela Olivas-Orozco, Carlos Horacio Acosta-Muñiz, Néstor Gutiérrez-Méndez, David Roberto Sepúlveda-Ahumada

**Affiliations:** 1 Centro de Investigación en Alimentación y Desarrollo, Cuauhtémoc, Chihuahua 31570, Mexico; nidia.chacon@estudiantes.ciad.mx (N.A.C.-F.); golivas@ciad.mx (G.I.O.-O.); cacosta@ciad.mx (C.H.A.-M.); 2 Facultad de Ciencias Químicas, Universidad Autónoma de Chihuahua, Chihuahua 31000, Mexico; ngutierrez@uach.mx

**Keywords:** water activity, lactic culture, *Escherichia coli*, bacterial inhibition, dairy products

## Abstract

This study aimed to evaluate the effectiveness of pH control, water activity (Aw), and the addition of lactic acid bacteria (LAB) on the proliferation of *Escherichia coli* in the curd during the manufacturing of Chihuahua cheese. Milk proved to be an excellent culture medium for *E. coli*, allowing it to develop at concentrations up to 10^9^ cfu/g. However, the presence of LAB, the pH control, Aw, and especially the use of the Cheddarization process during the Chihuahua cheese production proved to be important obstacles that inhibited the proliferation of *E. coli* under the conditions studied. Moreover, reducing the water activity of the curd as quickly as possible is presented as the most powerful tool to inhibit the development of *E. coli* during the Chihuahua cheese-making process.

## 1. Introduction

Consuming milk and dairy products is essential to the diet due to their composition rich in proteins, fats, lactose, mineral salts, and vitamins, among many other important nutrients [1]. This abundance of nutrients also offers optimal conditions for the growth of microorganisms, which can be beneficial or harmful to humans. As an example of beneficial bacteria, lactic acid bacteria (LAB) can be mentioned. These microorganisms are used in the food industry as starter cultures to obtain fermented dairy products [2]. Among the most used LAB genera are *Lactococus*, *Lactobacillus*, *Leuconostoc*, *Oenococcus*, and, within the genus *Streptococcus*, the species *Streptococcus thermophilus* [3]. On the other hand, there are microorganisms capable of causing illness in humans through food consumption, such as *Salmonella*, *E. coli*, *Listeria*, and *Campylobacter* [4]. Specifically, *E. coli* and some of its strains, such as *E. coli* O157:H7, are of great concern to the food industry (and a public health problem) due to their ability to cause serious diseases such as hemorrhagic colitis, hemolytic uremic syndrome, and thrombotic thrombocytopenic purpura [5]. Studies conducted in Canada and the United States have shown that *E. coli* ranks second among bacterial enteropathogens associated with non-specific diarrhea [6]. Recent studies conducted in Mexico establish that about 40% of fresh cheese from local retail markets show some degree of contamination with *E. coli* [7].

Chihuahua cheese, also known as Menonita cheese, is a traditional Mexican variety of cheese produced mainly in the state of Chihuahua, in the northern part of the country [8]. This cheese has a soft or semi-hard texture obtained by enzymatic coagulation of whole milk added with lactic acid bacteria. Once set, curds are cooked, cheddarized, salted, and pressed, obtaining in this way their characteristic attributes [9]. It is one of the main cheeses produced in Northern Mexico, a variant of young Cheddar cheese (2–4 weeks of maturation) and was developed by the Mennonite community that settled in the State of Chihuahua during the early years of the twentieth century [10]. According to the Mexican standard, the composition of Chihuahua cheese must include no more than 45% moisture, and at least 28% fat and 25% protein [9,11].

As part of the food manufacturing process, it is possible to manipulate some cheese attributes to inhibit the proliferation of microorganisms. Some of the most important processing factors include the control of water activity, the concentration of salt or other inhibitory substances, the oxidation-reduction potential, acidity, and temperature, as well as the addition of beneficial microorganisms and the modification of the atmosphere composition in which they are stored [12,13]. Several authors have studied modifying these factors to control the proliferation of *E. coli* during the manufacturing process of different cheese varieties. Previous studies have determined the effect of adding lactic cultures, antimicrobial substances, temperature control (during cooking, drainage, and storage), pH control, and storage conditions. These studies have determined that the proliferation of *E. coli* in milk and dairy products during manufacturing processes occurs in the early stages of processing, depending on the pH, temperature, and type of starter culture employed. Typically, once the population reaches high concentrations, it is difficult to inhibit its continued presence during storage [14,15,16,17,18,19,20,21,22,23,24].

Considering the results of these studies, the importance of inhibiting the proliferation of *E. coli* in the initial stages of the cheese-making process is evident. For this reason, the objective of the present study was to evaluate the ability of pH, water activity, and the presence or absence of lactic acid bacteria to act as obstacles in the proliferation of *E. coli* during the curdling of milk, which is the first stage in the cheese-making process.

## 2. Materials and Methods

**Figure 1:**
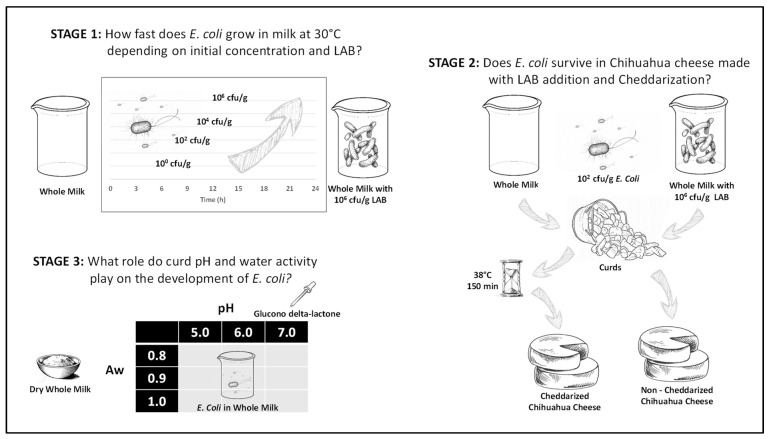
Experimental stages of the study.

The study was divided into three stages (Figure 1). The first part of the study characterizes the development of *E. coli* populations in liquid milk. The second part studies the development of *E. coli* throughout the Chihuahua cheese manufacturing process, and the third part of the study evaluates the influence of the water activity and pH of milk curds on the development of *E. coli* during the early stages of the Chihuahua cheese manufacturing processes.

During the first part of the study, the growth of *E. coli* in liquid milk was characterized to evaluate its proliferation under commonly found environmental conditions starting from four different initial *E. coli* concentrations. During this stage, the effect of the presence of a significant amount of LAB on the growth of *E. coli* in liquid milk was also evaluated.

Once *E. coli* growth in milk was characterized, a complete manufacturing process of Chihuahua cheese where slightly contaminated milk is used (100 *E. coli* cfu/g) was investigated. This initial *E. coli* concentration level is something that may be found in poorly controlled commercial processes, mainly due to post-pasteurization contamination or deficient pasteurization conditions. The ability of lactic acid bacteria to control the development of *E. coli* was evaluated on this second stage of the study, specifically in regard to the effectiveness of the cheddarization process, a stage where the LAB activity is promoted.

Finally, in the third stage of the study, a factorial experiment was designed to evaluate the joint effect of water activity and pH on the inhibition of *E. coli* in milk curds during the early steps of the manufacturing process in such a way that it was possible to determine the individual contribution of each factor on *E. coli* survival, as well as identifying possible interactions between them.

### 2.1. Bacterial Strains

In the present study, the *E. coli* strain ATCC 25922 and the lactic culture R-703 (CHR. HANSEN Laboratories, Orsholm, Denmark, containing *Lactococcus lactis* subsp. Lactis and *lactococcus lactis* subsp. *cremoris*) were used.

### 2.2. Preparation of E. coli and LAB Inocula

The *E. coli* strain was activated by adding a small portion of the lyophilized microorganism in LB Broth (Lennox, Sigma-Aldrich, St. Louis, MO, USA), incubating it at 37 °C for 24 h. Subsequently, an aliquot was sown in a petri dish (LB agar), and after incubation, a colony was transferred to a flask containing 50 mL of milk. It was then incubated at 37 °C while stirring at 180 rpm for 24 h until a final concentration of 10^9^ cfu/g was reached, corresponding to the early stationary stage of population development. In order to prepare the LAB inoculum, 5.8 mg of lactic culture *R-703* (CHR HANSEN containing *Lactococcus lactis* subsp. *lactis* and *Lactococcus lactis* subsp. *cremoris*) was weighed and added to 50 mL of milk and incubated at 37 °C with stirring at 180 rpm for 24 h until reaching a concentration of 10^8^ cfu/g.

### 2.3. Effect of the Initial Concentration on the Growth of E. coli in Milk

Commercial ultra-pasteurized milk was used to measure the development of *E. coli* populations in milk depending on its initial concentration. Milk samples were inoculated with four initial concentrations of *E. coli* ATCC 25922 (10^0^, 10^2^, 10^4^, and 10^6^ cfu/g) and were incubated at a constant temperature of 30 ° C for 30 h. Additionally, the development of *E. coli* populations was studied in milk previously inoculated with LAB at an initial concentration of 10^6^ cfu/g to evaluate the inhibitory capacity of LAB over *E. coli* popultations in liquid milk. The concentration of *E. coli* and LAB in milk, as well as the pH, were measured every 3 h. This incubation temperature was selected considering it is close to the temperature found in freshly milked non-refrigerated milk and coincides with the temperature used at the beginning of cheese manufacturing processes.

### 2.4. Effect of the Presence of LAB and Cheddarization on the Survival of E. coli in Chihuahua Cheese

Chihuahua cheese was made from pasteurized *E. coli*-free milk (63 °C for 30 min). After tempering milk at 30 °C, *E. coli* was added to obtain an initial concentration of 100 cfu/g. Next, the milk inoculated with *E. coli* was separated into two batches. One batch was added with LAB to obtain an initial concentration of 10^6^ cfu/g, while the other was processed without adding LAB as a control treatment. From this point, both batches were processed similarly by adding chymosin (CHY-MAX^®^ M, CHR Hansen, Orsholm, Denmark) in a proportion of 0.02% and resting for 30 min to allow curdling. After this stage, curd was cut into cubes of approximately 1 cm^3^ and cooked at 38 °C for 30 min. Once cooked, the curd was drained and separated once again into two batches: (1) Curd without cheddarization, which was immediately salted (2% sodium chloride), pressed, vacuum packaged in sterile bags, and stored at 4 °C, and (2) Curd subjected to cheddarization, which was dry incubated at 38 °C for 150 min before being salted, pressed, and packaged before storage at 4 °C. *E. coli* and LAB were quantified in liquid milk at the beginning of the manufacturing process and in the finished cheese after one week of refrigerated storage.

### 2.5. Effect of the pH and Water Activity on the Survival of E. coli in Curd

Milk curd conditions were simulated from commercial ultra-pasteurized milk standardized to 3 pH levels (7, 6, and 5) and three water activity levels (1.0, 0.9, 0.8) in a completely randomized factorial design resulting in nine treatments. Milk acidity was modified with glucono delta-lactone (Sigma-Aldrich), while water activity was controlled by the addition of low-heat whole milk powder. The development of *E. coli* was evaluated every 3 h for 24 h at 30 °C in each of the different treatments. Subsequently, the curd was stored in refrigeration at 4 °C for an extended period of four months to evaluate the survival or resurgence of *E. coli* under the conditions studied. The study of curds for an extended period of refrigerated storage was intended to determine whether the studied conditions (pH and Aw) were sufficient by themselves to ensure permanent control of *E. coli*. This extended storage period does not constitute a processing step in conventional Chihuahua cheese manufacturing processes.

### 2.6. Determination of pH and Aw

The pH measurement in milk, curd, and cheese was carried out using a potentiometer (HANNA instruments HI5221-01, Woonsocket, RI, USA) following the methodology described in the NOM-F-317-S-1978. On the other hand, water activity was determined using a standard laboratory water activity meter (AquaLab Series 3, Pullman, DC, USA).

### 2.7. Bacterial Quantification

Lactic acid bacteria were counted by the pour plate method using dilutions and culturing on sterile M17 agar (DIBICO, Cuautitlán Izcalli, México) with incubation in anaerobiosis for 24 h. Likewise, the pour plate method for coliform bacteria on Violet Red Bile Agar (Bioxon, Cd. México, México) with subsequent incubation at 37 °C for 24 h was used to determine coliforms [25].

### 2.8. Statistical Analysis

All experiments were performed in triplicate. The analysis of variance (ANOVA) was used to identify the main effects and interactions. In addition, Tukey’s range test was used to identify significant differences between means at a level of (*p* < 0.05). All statistical analyses were performed using Minitab 18 Statistical Software.

## 3. Results and Discussion

### 3.1. Development of E. coli in Milk from Four Different Initial Concentrations

**Figure 2:**
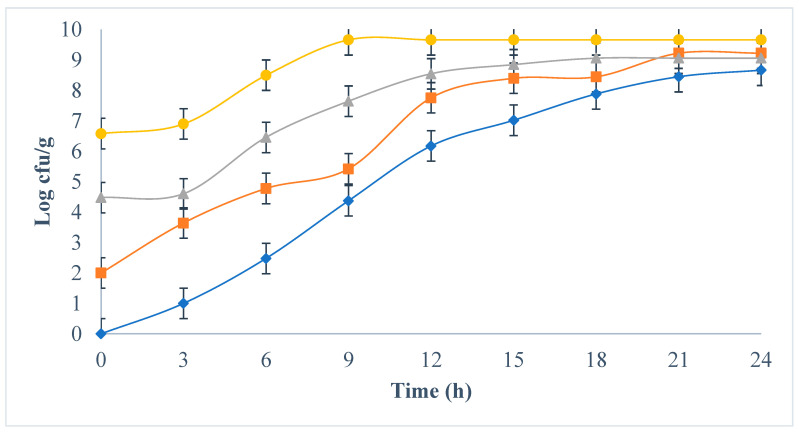
Development of *E. coli* in ultra-pasteurized milk incubated at 30 °C for 24 h, inoculated with four initial concentrations of *E. coli*: 10^0^ (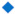), 10^2^ (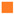), 10^4^ (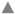), 10^6^ (●) cfu/g.

Figure 2 shows the development of *E. coli* in milk incubated at 30 °C. In all cases, *E. coli* showed sustained exponential growth until reaching a maximum value of approximately 10^9^ (stationary phase). The concentration of the initial *E. coli* inoculum played an essential role in defining the time required to reach the stationary phase being 24, 21, 15, and 6 h for treatments 10^0^, 10^2^, 10^4^, and 10^6^ cfu/g, respectively. These results confirm that milk is an excellent medium for the development and proliferation of *E. coli.* Similarly, the growth rate of *E. coli* provides a clear scenario of the risk involved in storing unprocessed or refrigerated milk, even when initial contamination levels are very low. Previous studies describe the proliferation of several *E. coli* strains during the production of cheese made from raw milk inoculated with *E. coli* (10^1^ and 10^3^ cfu/g), reporting an increase in *E. coli* counts during processing of approximately 3.5 decimal logs and finding significant differences between the strains studied, as well as between the initial *E. coli* inoculum levels studied [22,26].

**Figure 3:**
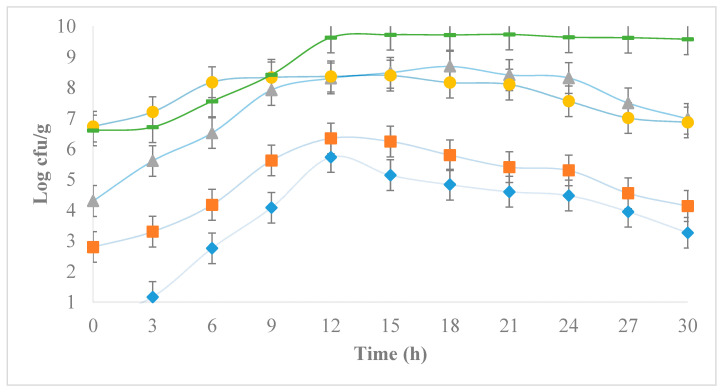
Development of *E. coli* in ultra-pasteurized milk inoculated with lactic acid bacteria 10^6^ cfu/g (**-**) incubated at 30 °C for 30 h, with four initial concentrations of *E. coli*: 10^0^ (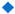), 10^2^ (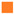), 10^4^ (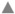), 10^6^ (●) cfu/g.

**Figure 4:**
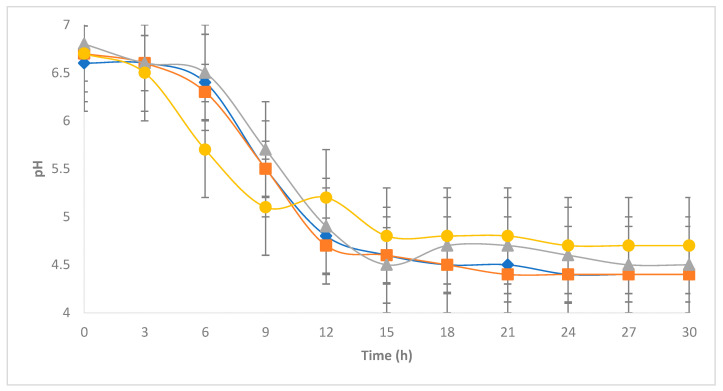
Development of pH in milk at 30 °C for 30 h. Milk was inoculated with a lactic culture (10^6^ cfu/g) and four initial concentrations of *E. coli*: 10^0^ (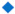), 10^2^ (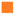), 10^4^ (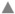), 10^6^ (●) cfu/g.

Figure 3 shows the development of *E. coli* incubated at 30 °C in milk previously inoculated with 10^6^ cfu/g of LAB. Similar to that observed in milk not inoculated with LAB, all treatments significantly increased the concentration of *E. coli* during the first 12 h of incubation, showing exponential growth. However, after 12 h, an inhibitory effect caused by the presence of LAB can be observed, which likely limited the development of *E. coli* through pH reduction (Figure 4), competition for nutrients, and synthesis of lactic acid or some other bacteriostatic substance [15], preventing all treatments from reaching the maximum concentration of 10^9^ cfu/g observed in milk without LAB. In the case of milk added with LAB, the maximum levels of *E. coli* proliferation observed were 10^5^, 10^6^, 10^8^, and 10^8^ cfu/g for treatments with initial *E. coli* concentrations of 10^0^, 10^2^, 10^4^, and 10^6^ cfu/g, respectively. These observed maximum concentrations of *E. coli* were further reduced during the remaining 18 h of monitoring, reaching final values of 10^3^, 10^4^, 10^6^, and 10^6^ cfu/g, respectively, implying an additional reduction of about two logarithmic cycles caused by the presence of LAB. Although promising, the inhibitory effect of LAB observed in this study cannot be considered sufficient from a public health standpoint since the final concentrations of *E. coli* are at considerably high levels in all treatments despite the observed reduction. Previous studies evaluating the ability of LAB to inhibit the development of *E. coli* in liquid milk demonstrate practically null control after *E. coli* populations exceed 10^4^ cfu/g [27]. Inhibitory effects on other pathogens, such as *Salmonella* spp., observed as a result from the pH decrease caused by LAB have been previously reported, emphasizing that the type of chemical species responsible for acidification may play a vital role in defining the intensity of the observed effect [28]. The relative abundance of protonated lactic acid in particular, rather than pH suppression itself, has been reported as a critical factor defining the effectiveness of LAB in reducing *E. coli* populations [29].

### 3.2. Development of E. coli during the Chihuahua Cheese-Making Process

**Figure 5:**
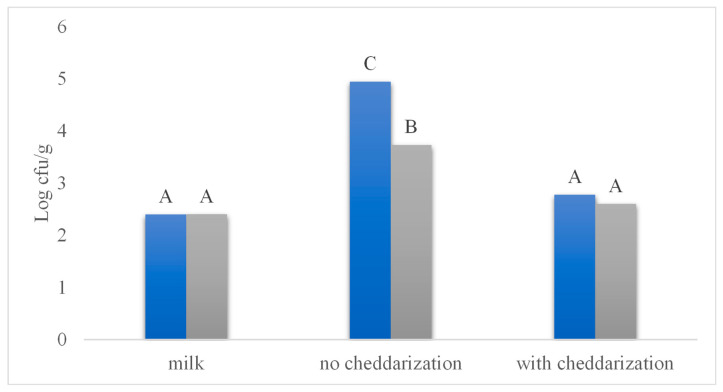
Concentration of *E. coli* in Chihuahua cheese manufactured without the addition of Lactic Acid Bacteria (blue bars) and with addition of lactic acid bacteria (gray bars). *E. coli* concentration was determined on milk prior to cheese manufacture, on finished Chihuahua cheese in which no cheddarization was conducted, and on finished Chihuahua cheese that was manufactured employing the cheddarization processing step. Bars with the same superscript letter are not statistically different.

Figure 5 shows the development of the *E. coli* population in the different Chihuahua cheese production processes studied. The initial concentration of *E. coli* of 10^2^ cfu/g increased rapidly during the curdling, cooking, and draining stages, reaching a maximum level at the end of the process of 10^5^ cfu/g in the cheese without LAB addition or cheddarization. A cheese with this high concentration of *E. coli* undoubtedly poses a health risk. An increased concentration of *E. coli* during the cheese manufacturing process, starting from the draining stage has been previously reported, indicating that bacterial cells tend to multiply and remain trapped in the three-dimensional casein structure while whey is expelled in the later stages of the process [30]. However, it is crucial to note that, although this treatment (no LAB addition, no Cheddarization) did not experience a very drastic change in pH (6.2), given the absence of LAB inoculum, the *E. coli* population did not reach levels of 10^8^ cfu/g as would have happened in the case of liquid milk (pH 6.7) after 12 h of elapsed time (Figure 2). This indicates the presence of a preservation effect inherent to the manufacturing process, possibly related to salt addition and water activity reduction [13]. It is also important to consider that pasteurized milk (such as the one used in this study) has a natural microbiota of non-pathogenic microorganisms in a concentration in the order of 10^2^ cfu/g [31]. The presence of these microorganisms may also have played a role in the observed inhibitory effect, although this effect is probably marginal given their low concentration.

On the other hand, cheese made without cheddarization but from milk added with LAB showed a less pronounced increase in the concentration of *E. coli*, reaching a maximum concentration at the end of the process of 10^3^ cfu/g, which implies a difference of two logarithmic cycles compared to treatment without LAB addition, and an increase of only one logarithmic cycle compared to the initial concentration of *E. coli* in milk. This greater inhibitory power of cheese inoculated with LAB can undoubtedly be related to the pH decrease (5.5), the synthesis of organic acids, and possibly other bacteriostatic substances, as well as to the competitive inhibition caused by the presence of a high LAB concentration from the beginning of the process [32]. Previous studies [33] have demonstrated the ability of LAB, such as *Lactococcus lactis* subsp. *lactis* biovar. diacetylactis, to inhibit the development of pathogenic microorganisms such as *E. coli* and *Salmonella enteritidis*, basing its effectiveness on the rapid production of lactic acid, and the consequent rapid pH decrease.

Finally, the cheddarization process proved to be the most relevant stage of the Chihuahua cheese manufacturing process in terms of limiting the development of *E. coli.* Figure 5 shows how the cheese made from milk inoculated with LAB and the one manufactured without adding LAB benefit both from an incubation period at 38 °C for 150 min after draining the curd and before salting and pressing. The results of this study demonstrate how cheddarization generates conditions that promote the proliferation of beneficial microorganisms, even in cases where a large amount of specific LAB has not been added at the beginning of the process. All cheddarized cheeses studied maintained their *E. coli* population within the original inoculum level of 10^2^ cfu/g, showing an effective bacteriostatic effect. Although the treatment with the addition of LAB and cheddarization shows an apparently lower concentration of *E. coli* than the cheddarized treatment without the addition of LAB, both were statistically indistinguishable compared to the concentration of *E. coli* in milk at the beginning of the process. As mentioned before, the pasteurized milk used in this study contains limited amounts of LAB, bacteria capable of surviving the pasteurization process, which take advantage of the incubation conditions provided by cheddarization, matching the effect obtained with the initial addition of high concentrations of LAB. Previous studies conducted on raw and pasteurized milk artificially contaminated with different *E. coli* strains during refrigerated storage showed a reduced concentration of *E. coli* after the second day of cold storage in non-pasteurized milk, which showed a much larger populations of LAB and total aerobic plate count than pasteurized milk by that time of the refrigerated storage [34]. In addition, cheddarization favors the decrease of Aw and pH, both critical factors on the inhibition of undesirable microorganisms. As pH decreases below 5.4, the growth of gas-forming organisms such as coliforms markedly decreases [35].

### 3.3. Effect of pH and Water Activity on the Growth of E. coli in Simulated Curds

**Figure 6:**
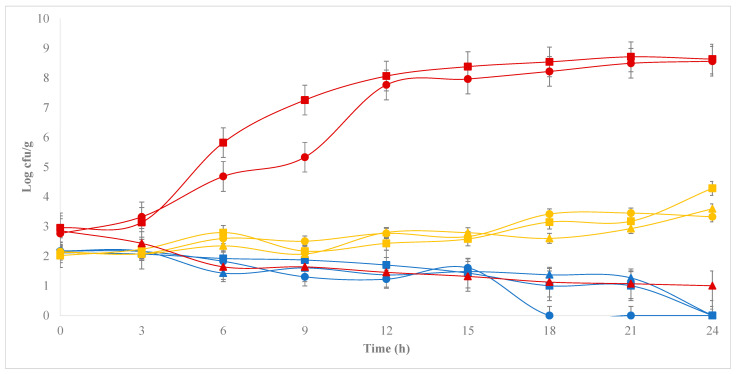
*E. coli* concentration in simulated curds standardized to three pH levels and three water activity (Aw) levels monitored for 24 h at 30 °C. Red lines correspond to treatments of Aw 0.8, yellow lines to Aw 0.9, and blue lines to Aw 1.0. Round symbols represent pH 7.0 treatments, pH 6.0 treatments are represented by squares, and pH 5.0 treatments are represented by triangles.

Figure 6 shows the development of *E. coli* in simulated curds monitored for 24 h at 30 °C with different combinations of pH and water activity. Statistical analysis of the data revealed a significant effect (*p* < 0.05) of water activity and of the interaction between pH and water activity. Water activity proved to be the most relevant factor defining E. *coli* growth. While simulated curds adjusted to 0.8 water activity show the ability to inhibit *E. coli* growth within the 24 h studied, curds with water activity of 0.9 showed only bacteriostatic capacity. Finally, curd with water activity of 1.0 allows the growth of *E. coli* without any restriction, showing a behavior similar to that observed in liquid milk (Figure 2). pH control through glucono delta-lactone addition, on the other hand, does not seem to cause a relevant effect on the development of *E. coli* under the conditions studied, except for the treatment of water activity 1.0 and pH 5, which showed significant *E. coli* inhibition capacity, attributable entirely to the low pH employed. Results similar to those obtained in the present study, suggesting interactions between pH and water activity in controlling *E. coli* growth, have been reported before [36].

The follow-up of the *E. coli* population in the simulated curds studied during extended storage at 4 °C (Table 1) revealed that treatments with a water activity of 0.8 effectively eradicated the *E. coli* population presenting “Undetectable” counts at day 1, 63, and 126 of extended storage, regardless of the pH employed. Treatments with 0.9 water activity, on the other hand, showed a continuous bacteriostatic capacity, presenting counts in the order of 10^3^ cfu/g still on day 1 of storage, which were reduced to 10^2^ cfu/g after 63 days, reaching levels of “Non-Detectable” at 126 days of extended refrigerated storage. Finally, curds with a water activity of 1.0 maintained high concentrations of *E. coli* throughout the extended storage period studied, presenting concentrations of 10^8^, 10^5^, and 10^4^ cfu/g after 1, 63, and 126 days, respectively. As mentioned before, the treatment of water activity 1.0 and pH 5.0 showed a different behavior than other treatments with water activity 1.0, presenting total growth inhibition of *E. coli* from day 63 to 126. Previous studies have reported the survival of *E. coli* in various cheese varieties for extended storage times. For example, Schlesser, Gerdes [19] (2006) report survival of *E. coli* for 60 days in Cheddar cheese made with unpasteurized milk, inoculated with a cocktail of five *E. coli* strains at an initial concentration of 10^5^, 10^3^, 10^1^ cfu/g and stored at 7 °C. Something similar was observed in Cheddar cheese made with raw and pasteurized milk artificially enriched with 10^5^, 10^3^, and 10^1^ cfu/g of *E. coli* O157:H7 stored at 15 °C. In this case, although coliform bacteria naturally present in raw milk were completely inactivated at five weeks of storage, artificially inoculated *E. coli* was able to survive the entire 60-day storage period, suggesting that the maturation process alone is not sufficient to ensure complete inactivation of contaminating pathogens [37]. Finally, a study conducted on Gouda and Cheddar cheeses made from raw milk contaminated with *E. coli* O157:H7 at a level of approximately 20 cfu/g and stored at 9 °C showed survival after 60 days at average levels of 25 and 5 cfu/g in Cheddar and Gouda, respectively, observing detectable levels of *E. coli* after selective enrichment for more than 270 days in both types of cheese [30].

## 4. Conclusions

The present study demonstrates that the inoculation of LAB in milk prior to cheese manufacture may be capable of moderately controlling the development of *E. coli* populations during Chihuahua cheese manufacturing. However, this effect is limited at its best to a bacteriostatic effect dependent on the initial concentration of *E. coli*. By itself, LAB inoculation can hardly be considered as a satisfactory means to eradicate *E. coli* of cheese completely.

The use of the cheddarization step was shown to be a relevant strategy to deter the increase of the population of *E. coli* during the Chihuahua cheese manufacture process. This observation results relevant as the cheddarization process is sometimes considered only as a texturing step in which the development of casein fibers modifies the physical structure of cheese, hence disregarding it as a relevant unit operation from a food safety point of view. The results of the present study demonstrate that cheddarization is capable of controlling the increase of the *E. coli* population even when no LAB starters are added to milk. Therefore, the cheddarization step becomes an essential step to reduce the risk of *E. coli* presence in Chihuahua cheeses that are produced in artisanal manufacture processes that rely exclusively on the presence of native LAB for the development of acidity in the manufacture process.

Finally, this study demonstrated the importance of reducing the water activity of curds as much as possible during the cheese manufacturing process, especially in the early steps of curdling and cooking, since this parameter turned out to be the most critical in defining the survival of *E. coli* in Chihuahua cheese.

## Figures and Tables

**Table 1:** Growth of *E. coli* (cfu/g) in simulated curds during extended storage at 4 °C.

Aw	pH	Day 1	Day 63	Day 126
0.8	7.0	ND	ND	ND
0.8	6.0	ND	ND	ND
0.8	5.0	ND	ND	ND
0.9	7.0	2.10 × 10^3^	6.60 × 10^2^	ND
0.9	6.0	1.91 × 10^3^	1.50 × 10^2^	ND
0.9	5.0	3.98 × 10^3^	1.80 × 10^2^	ND
1.0	7.0	3.60 × 10^8^	9.00 × 10^5^	5.30 × 10^4^
1.0	6.0	4.30 × 10^8^	2.90 × 10^5^	1.00 × 10^4^
1.0	5.0	1.00 × 10	ND	ND

ND, not detected.

## Data Availability

Data is contained within the article.
